# Treatment of Severe Infections Due to Metallo-Betalactamases *Enterobacterales* in Critically Ill Patients

**DOI:** 10.3390/antibiotics11020144

**Published:** 2022-01-24

**Authors:** Jean-François Timsit, Paul-Henri Wicky, Etienne de Montmollin

**Affiliations:** 1 APHP, Service de Médecine Intensive et Réanimation Infectieuse, Hôpital Bichat Claude-Bernard, 46 Rue Henri Huchard, 75018 Paris, France; jean-francois.timsit@u-paris-sante.fr (P.-H.W.); etienne.demontmollin@aphp.fr (E.d.M.); 2 IAME, INSERM, University of Paris, 75018 Paris, France

**Keywords:** metallo-beta-lactamases, sepsis, critically ill, aztreonam, cefiderocol, avibactam, NDM, VIM, pneumonia, bloodstream infections

## Abstract

Metallo-beta-lactamases-producing (MBL) *Enterobacterales* is a growing problem worldwide. The optimization of antibiotic therapy is challenging. The pivotal available therapeutic options are either the combination of ceftazidime/avibactam and aztreonam or cefiderocol. Colistin, fosfomycin, tetracyclines and aminoglycosides are also frequently effective in vitro, but are associated with less bactericidal activity or more toxicity. Prior to the availability of antibiotic susceptibility testing, severe infections should be treated with a combination therapy. A careful optimization of the pharmacokinetic/pharmacodynamic properties of antimicrobials is instrumental in severe infections. The rules of antibiotic therapy are also reported and discussed. To conclude, treatment of severe MBL infections in critically ill patients is difficult. It should be individualized with a close collaboration of intensivists with microbiologists, pharmacists and infection control practitioners.

## 1. Introduction

Carbapenem-resistant *Enterobacterales* (CRE) is a growing problem worldwide [1,2,3,4,5,6]. Carbapenem resistance in *Enterobacterales* is mostly due to carbapenemases. Carbapenemases are characterized as either metallo-beta lactamases (MBL) in Ambler class B or serine beta-lactamases in Ambler class A or D. MBL can inactivate all bi-cyclic beta-lactams and serine beta-lactamase inhibitors that are used in human medicine, such as sulbactam, tazobactam, clavulanic acid, avibactam and vaborbactam. Class B1 enzymes includes Verona integron-encoded MBLs (VIM), imipenemases (IMP) and New Delhi MBL (NDM). MBL per se cannot hydrolyze monobactams.

The rapid spread of MBLs worldwide is responsible for life-threatening infections which are particularly difficult to treat given the paucity of active available antimicrobials. In this review, we will focus on the optimization of treatment of MBLs infections in critically ill patients.

## 2. Trends in Epidemiology

MBLs have spread globally within *Enterobacterales* in the past decade. CRE rates increased by more than 2.5-fold from 2013 to 2020. Globally, it represents one fifth of the carbapenem-resistant *Enterobacterales* isolated from clinical samples [2,7]. NDM represents more than half of the MBL. The rapid spread of the *blaNDM* gene may partly be due to a limited fitness cost of this enzyme to *Enterobacterales* [8]. There is important variability of the rate of MBL producing *Enterobacterales* among CRE between regions. MBLs represent less than 6% of the carbapenem-resistant *Enterobacterales* in North and Latin America, but more than 40% in the Middle East, Africa and Asia/South Pacific [2]. In Europe, VIM represents about 8% of the CRE, mainly in Southern Europe. NDM has spread to all European countries and represents more than 7% of CRE.

## 3. Risk Factors

Risk factors of MBL infections are similar to the risk factors of infections with other CREs. These risk factors include prior colonization, prior antimicrobial use, healthcare exposure, comorbidities, ICU admission, mechanical ventilation, dialysis and the presence of indwelling catheters [9]. Snyder et al. performed a retrospective case–control study in India to identify risk factors of bloodstream infections (BSIs) caused by NDM-1-producing strains. As compared to BSI due to other multidrug-resistant strains, prior carbapenem use (OR 8.4) and central venous catheter (OR 4.8) predicted the acquisition of an NDM-1 strain [10].

The risk of mortality associated with MBL *Enterobacterales* infections is considerable. In a study performed in Athens, one third of the patients died within 14 days following infections with VIM-positive *Klebsiella pneumoniae* [11]. Similar mortality was observed in India and South Africa in patients with bloodstream infections (BSI) due to NDM-producing *Enterobacterales* [10,12]. Two recent studies suggested that prognosis recently improved with the growing use of active beta-lactams antibiotics. In a multicenter study including 102 BSI episodes in Italy and Greece, 30-day mortality was 31.4% [13]. Of 57 nosocomial infections due to NDM-producing bacteria in India (72% in ICU), 30-day mortality was 21% [14].

## 4. From Empirical to Early Documented Therapy

In critically ill patients, antibiotic therapy must be immediately effective on the pathogens [15]. In routine practice, the decision to start treatment active against MBL is based on the answers to important questions [16].

The risk of MBL infection will depend on local epidemiology and the history of recent MBL outbreaks [17]. Previous colonization markedly amplifies the risk of subsequent infection with MBL and is a key component of an empirical therapy [18,19]. However, the positive predictive value of this risk factor is low [17]. In one study, the rates of infection among carbapenemase-producing *Enterobacterales* carriers were higher for KPC-producing (60%) than for NDM-producing *Enterobacterales* (12%) [20].

The risk of MBL infections is higher in patients with advanced co-morbid illnesses, prolonged hospital stays, and who had undergone invasive procedures [21]. Prior carbapenem exposure in the past 30 days is also a risk factor of MBL infection, but to a lesser extent than the risk of non-carbapenemase-producing carbapenem-resistant *Enterobacterales* [22].

Rapid molecular diagnostic tests are increasingly being developed to identify pathogens and antibiotic resistance patterns, but are expensive and not available everywhere [23,24]. They still require time for sample collection, lab delivery, and specimen analysis, and during this time, antibiotic therapy is usually not withheld.

There is probably room for developing artificial intelligence or machine-learning to help to bridge this time gap, e.g., by predicting antimicrobial resistance patterns. The first attempts at predicting carbapenem resistance provided encouraging results. In a recent analysis, McGuire and colleagues demonstrated that longitudinal clinical data could predict the risk of carbapenem resistance [25]. In this investigation, new carbapenem-resistant infections accounted for 1.6% of the population, yet the predictive model generated a sensitivity of 30%, a positive predictive value of 30% and a negative predictive value of 99% (AUROC 0.84).

## 5. Available Drugs

### 5.1. Ceftazidime-Avibactam/Aztreonam

MBL can hydrolyze all beta-lactams except aztreonam (ATM). However, in MBL-producing *Enterobacterales*, aztreonam is frequently hydrolyzed by other beta-lactamases that are frequently co-produced. In total, ATM alone remains active in no more than one third of MBL isolates. The combination of ATM and a beta-lactam/beta-lactamase inhibitor such as ceftazidime avibactam (CZA) restores the intrinsic activity of ATM on MBL and is an attractive option for therapy. Indeed, CZA is active against Ambler A, C and D beta-lactamases including extended spectrum beta-lactamases such as CTX-M, AmpC and OXA-48. In a systematic review of in vitro data, MIC values ≤ 4 mg/L for ATM in combination with CZA have been described in 79.6% of the MBL-producing *Enterobacterales* [26].

The association was tested as a last-resort therapy and reported in a growing number of case-reports and cohorts [26]. In a multicenter cohort of 102 patients with BSI due to MBL-producing *Enterobacterales* (NDM *n* = 82, VIM *n* = 20), Falcone et al. compared the activity of CZA/ATM with other various combination therapies [27]. Of the 52 patients that received CZA/ATM, half were in ICU, 26% had septic shock and 30% received mechanical ventilation. The source of BSI was mainly urine (32%) and intravascular catheters (26.5%). Ten out of 52 died (19.2%), while clinical failure at day 14 was only diagnosed in 13 (25%) of them. After adjustment on SOFA score, chronic diseases, CZA/ATM use was associated with an improved survival rate (Hazard ratio 0.17 [95% Confidence Interval, 0.07–0.41]; *p* < 0.001). Among the patients not treated with CZA/ATM, the highest 30-day mortality was observed in patients treated with colistin-based regimens (59.3%).

Nagvekar et al. [14] reported 40 cases of severe infections due to *Enterobacterales* (*Klebsiella n* = 53, *Escherichia coli n* = 26) that carried either NDM alone or the combination of OXA-48 and NDM. The source of infection was intra-abdominal (32%), ventilator-associated pneumonia (26%), complicated urinary tract infections (9%) and bloodstream infections (9%). Seventy-two percent of the cases were hospitalized in ICU. CZA/ATM alone (*n* = 12) resulted in 11 clinical cure (92%). Combination with colistin (*n* = 21) or fosfomycin (*n* = 7) was associated with a clinical cure rate of 20 (71.4%).

We recently published the cases of two organ transplant recipients with septic shock due to NDM1-*Klebsiella pneumoniae* with ventilator-associated pneumonia and bloodstream infection; the clinical success was obtained after a 14-day CZA/ATM therapy [28]. In both cases, recurrences occurred within 30 days and were microbiologically and clinically controlled with the same antimicrobials. One of the patients subsequently died due to transplant rejection.

We treated nine patients with the combination therapy within the past 2 years for NDM-producing *Enterobacterales* in our ICU (Table 1), either after documentation or using the results of the multiplex polymerase chain reaction (mPCR) on respiratory secretions and previous known colonization. Infection was confirmed in seven cases (VAP, four; BSI of unknown origin, one; peritonitis, one; surgical site infection, one). The MIC of CZA/ATM was lower than 0.5 mg/L in all cases. Microbiological eradication was obtained in all but one case, and clinical cure was obtained in five out of seven cases. Four out of seven patients died; the death was probably related to NDM infection for two of them, and definitely unrelated for the remaining two others.

Even if clinical data are encouraging, many questions remain. The optimal dose of CZA/ATM is unknown. IDSA [29] recommends ceftazidime-avibactam 2.5 g IV q8h, infused over 3 h plus aztreonam: 2 g IV q8h, infused over 3 h, according to previous clinical studies [27]. A recent simulation model on hollow-fiber suggests that CZA 2 g every 8 h and ATM 2 g every 6 h over 2 h, or both agents administered in continuous infusions, yielded better bacterial killing with no emergence of resistance within 7 days [30]. Importantly, ATM and CZA should be given simultaneously. The combination of aztreonam and avibactam (AVI) is currently on phase III of the development process. The proposed dose is 500 mg ATM/167 mg AVI loading dose within 30 min followed by 1500 mg ATM/500 mg AVI over 3 h IV every 6 h [31].

### 5.2. Cefiderocol

Cefiderocol is a novel siderophore cephalosporin with unique broad-spectrum activity and stability against all classes of carbapenemases, (KPC, OXA, NDM, VIM and IMP). It enters the bacterial cell through the iron transporters, shunting the need for porin channels. It is stable for hydrolysis by various beta-lactamases including MBLs. Using a breakpoint of 4 mg/L, MBL-producing *Enterobacterales* are susceptible to more than 72% of NDM producers, 91.7% of VIM producers and 87% of IMP producers. However, for NDM producers, the MIC_50_ is 1 to 4 mg/L, i.e., very close to the breakpoint. Furthermore, it should be known that different testing modalities may lead to subsequent variation of MIC measurements in *Enterobacterales* [32].

In the CREDIBLE-CR [33] and APEKS-NP [34] studies, cefiderocol monotherapy was effective against Gram-negative bacteria producing metallo-beta-lactamases. Overall, rates of clinical cure (70.8% (17/24)), microbiological eradication (58.3% (14/24)), and 28-day all-cause mortality (12.5% [3/24]) compared favorably with comparators of best available therapy and high-dose meropenem (40.0% (4/10); 30.0% (3/10); and 50.0% (5/10)), respectively. Clinical cure was lower for NDM (9/16, 56.2%) than for non-NDM (8/8, 100%) infections.

In an in vitro evolution experiment using clinical NDM-*Enterobacter cloacae* isolates via serial passaging, cefiderocol pressure leads to resistance acquisition. It was suggested that the presence of NDM facilitates the emergence of resistance via non-synonymous mutations of the CirA catecholate siderophore receptor [35].

In vivo acquired resistance to cefiderocol has been already reported [36] due to an increase in the copies of *blaNDM5* gene of *E. coli,* resulting in a clinical failure to treat an intra-abdominal infection, which was eventually successfully treated by a combination of CZA/ATM. 

To conclude, if a metallo-beta-lactamase (i.e., NDM, VIM, or IMP) is identified, preferred antibiotic options include ceftazidime-avibactam plus aztreonam, or cefiderocol monotherapy. Clinical outcome data comparing these two treatment strategies are not available [29,37].

### 5.3. Carbapenems

In vitro activity of carbapenem has been reported in up to 60% of VIM-positive *Enterobacterales* [38]. However, CRE with borderline susceptibility to carbapenem exhibits a marked inoculum effect, with a more than 8-fold increase in the MIC for inoculums between 10^4^ and 10^5−6^ cfu/mL [39]. The use of carbapenems monotherapy in carbapenemases-producing *Klebsiella pneumoniae* is limited, and resulted in a rate of clinical failure of 25% [40]. It should be discussed when meropenem MIC is ≤8 mg/L [37]. Data on carbapenems use in MBL infections are limited [41]. Considering the in vitro data, the absence of strong clinical evidence, and the available alternative antibiotics, the use of carbapenem in susceptible strains should be discouraged [29].

### 5.4. Tetracyclines

Tetracyclines such as tigecycline are active against most cases of *blaNDM Enterobacterales* [42], but stable plasmidic resistance has been described. Tigecycline also remains active in vitro against most parts of other MBL-producing *Enterobacterales* [43,44]. One animal model suggested that tigecycline alone at 50 mg bid or 100 mg bid is not sufficient to control pneumonia due to NDM-producing *Enterobacterales* [45]. Indeed, administered in monotherapy at such humanized doses, it resulted in bacterial regrowth.

### 5.5. Fosfomycin

Fosfomycin is a broad-spectrum antimicrobial. It is active against *Enterobacterales* including MBL producers [46], but with a high risk of acquired resistance. Fosfomycin diffusion in body tissues is excellent. In a neutropenic murine thigh infection model due to NDM *K. pneumoniae*, the AUC/MIC ratio needed to achieve one log kill was 22 [47]. It is also active against more than 90% of MBL-producing *Enterobacterales* [48,49]. With a dose of 6 g IV every 8 h, the AUC is about 715 mg.h/L [50]. Therefore, bacterial killing should be obtained if MICs are not higher than 8 mg/L. Monotherapy with fosfomycin should not be used to treat MBL-producing *Enterobacterales* infections, due to baseline hetero-resistance and frequently observed regrowth. The place of fosfomycin, always in combination therapy, especially to optimize the treatment of infected tissues, remains to be evaluated. Fosfomycin may display synergy with carbapenems and/or colistin against NDM-producing *K. pneumoniae,* but resistance via metallo-enzymes has been described and the intravenous formulation is not available in the U.S.

### 5.6. Polymixins

Polymixins are active in more than 90% of the cases against MBL-producing *Enterobacterales,* with an MIC90 of 1 mg/L [48]. It should be kept in mind that it is naturally inactive against *Proteus*, *Morganella*, *Providencia* and *Serratia* spp. It was one of the pivotal drugs used for treating MBL infections before 2015 [51]. In ICU, intravenous colistin should be given at high doses (i.e., 75 à 150,000 U/Kg/d with a maximal dose of 12 MUI per day). The therapeutic margin of colistin is narrow, and high concentrations are associated with increased renal and neurological toxicity. In the past few years, many studies have suggested not using colistin in difficult-to-treat Gram-negative infections when an alternative exists. A colistin-based regimen was associated with an increased risk of acute kidney injury [52,53,54,55,56]. In a systematic review, Wagenthaler et al. estimated the rate of nephrotoxicity of polymixins to be as high as 39% [57]. The odds of nephrotoxicity were greater with polymyxin-based therapies compared to non-polymyxin-based regimens (odds ratio 2.23 (95% CI 1.58–3.15); *p* < 0.001). Cohort studies suggest that colistin monotherapy is less effective than in combination in treating CRE infections [58,59]. 

### 5.7. Aminoglycosides

Aminoglycosides are rapidly bactericidal. Resistance, due to aminoglycosides-modifying enzymes, is common in MBL strains [60]. In a recent study from Greece, MBL-producing *Enterobacterales* were susceptible to gentamicin in one-third of the strains, but rarely susceptible to amikacin. Plazomycin is able to evade to enzymes and is active in more than 80% of the VIM and around half of the NDM-producing *Enterobacterales* [44,60,61]. The drug has been successfully tested in combination in CRE infections (mainly KPC) [62], and was approved by the FDA in 2018. Unfortunately, it is not commercialized yet. Overall, the high resistance rate precludes the use of aminoglycosides as empiric therapy. It may be used in association with other antibiotic therapies based on documented infections.

## 6. A Place for Nebulized Antimicrobials in MBL-Producing *Enterobacterales* Pneumonia

Despite appropriate parenteral antimicrobial therapy, VAP remains associated with a substantial risk of therapeutic failure. Potential causes of failure are a high bacterial inoculum, poor lung diffusion of antibacterial agents, reduced bronchial bacterial clearance by the alteration of the mucus layer, altered bacterial mechanical clearance and impaired local immunity. Of course, the situation is even more complex when pathogens are poorly susceptible to available antibacterial agents and when the minimum inhibitory concentration (MIC) is close to or beyond the resistance breakpoint. Nebulization of antimicrobials is feasible and widely used [63] in mechanically ventilated patients. It allows the delivery of extremely high concentrations of antimicrobials directly to the targeted tissue. It is especially interesting for MBL-producing organisms and for molecules with reduced lung diffusion and high dose-dependent systemic toxicity when administered parenterally, such as aminoglycosides and polymixins.

It should be kept in mind that the ECCMID task force recommends avoiding nebulized antimicrobials in patients with severe hypoxemia (PaO2/fiO2 < 200 mmHg) or in patients that have shown signs of poor pulmonary reserve or tending to rapid lung derecruitment. This condition is frequent in all patients with moderate to severe acute respiratory distress syndrome [64].

In a recent meta-analysis of cohort studies, the use of adjunctive nebulized antibiotics in VAP improved the rates of clinical cure (relative risk (RR) 1.13, 95% CI (1.02, 1.26)) and microbiological eradication (RR 1.45, 95% CI (1.19, 1.76)) but had no impact on mortality (RR 1.00, 95% CI (0.82, 1.21)) [65]. Inhaled antibiotic therapy was associated with an increased risk of bronchospasm (RR 2.74, 95% CI (1.31–5.73)) [65]. Adjunctive nebulized antibiotics had no effect on the duration of mechanical ventilation and on the ICU length of stay.

A single-center double-blind trial compared an adjunctive therapy of 7 days of aerosolized amikacin (400 mg tid) versus placebo administered via a jet nebulizer on VAP due to resistant Gram-negative bacteria (*Acinetobacter baumannii n* = 16, *P. aeruginosa n* = 15, *Enterobacterales* (*n* = 22) and other non-fermentative bacteria (*n* = 7)). Adjunctive nebulized antibiotic resulted in a quicker clinical improvement without effects on the delay in successful ventilator weaning or 28-day mortality. Bacterial eradication was more frequently obtained at the end of treatment with adjunctive nebulized amikacin (13/32 vs. 4/28, *p* = 0.024) without the emergence of amikacin resistance during the 28-day follow up [66]. Two other randomized double-blind studies, using adjunctive inhaled amikacin combined with fosfomycin [67] or amikacin [68], also suggested that adjunctive inhale antibiotics may lead to a higher bacterial eradication in extensively and pan-drug resistant Gram-negative pneumonia with no significant impact on clinical cure or mortality.

A positive effect of adjunctive antimicrobial nebulization was also suggested by a single-center double-blind RCT in chronically intubated critically ill patients at risk of infections with multidrug-resistant organisms (MDRO). Inhaled antibiotics for 14 days (mainly aminoglycosides) resulted in more bacterial eradication at the end of treatment (14 out of 16 patients compared with 1 of 11 for placebo (*p* < 0.001)). New resistance was less common when an adjunctive inhaled antibiotic was used (2/16 vs. 6/11, *p* = 0.03) [69].

In the IDSA guidelines, for patients with VAP due to Gram-negative bacilli that are susceptible to only aminoglycosides or polymyxins (colistin or polymyxin B), the experts suggested combining both inhaled and systemic antibiotics, rather than systemic antibiotics alone (weak recommendation, very low-quality evidence). It is reasonable to consider adjunctive inhaled antibiotic therapy as a last-resort treatment for patients who are not responding to intravenous antibiotics alone, whether the infecting organism is a MDRO or not [70].

Importantly, the small benefit observed in bacterial clearance of MDR/PDR GNB does not automatically translate into improved clinical outcomes and may be counterbalanced by poor tolerance, especially in patients with severe alteration of oxygenation. Its use should be limited to units experienced in nebulization, using checklists and specific surveillance [71] in order to reduce the risk of misuse and adverse effects.

## 7. Monotherapy or Combination in Critically Ill Patients

There are some indirect data suggesting that combination therapy is preferable for severe infections due to CRE. This is especially the case when strains are not treated with new beta-lactams antimicrobials [72]. The INCREMENT cohort compared monoactive and dual active antibiotic therapy for CRE (almost exclusively KPC and OXA-48) [59]. In the most severe patients, combination therapy was associated with lower mortality than monotherapy was (30 (48%) of 63 vs. 64 (62%) of 103; adjusted HR 0·56 (0.34–0.91); *p* = 0.02). It should be kept in mind that available clinical data on this topic are scarce and associated with important limitations. Indeed, randomized control trials have never been performed. Published cohort studies only referred to targeted therapy and did not consider the potential benefit of a combination therapy for treatment started empirically, leading to an important risk of immortal time bias.

Moreover, in infections due to MBL-producing strains, the presence of co-existing resistance mechanisms leaves very few therapeutic options for combinations.

The combination of a pivotal beta-lactam and of one of the possible therapeutic alternatives is not usually recommended for MBL infections [37,73]. However, combination therapy might be considered for the empirical therapy of patients previously colonized with MBL producers. Combination may also be considered when the initial bacterial inoculum is very high, such as in hospital-acquired pneumonia and VAP. In pneumonia, if there is no severe hypoxemia, nebulization of colistin of aminoglycosides might be considered.

## 8. Therapeutic Rules

Some rules should be taken into account when deciding and implementing the therapy of patients with suspected or proven MBL infections [16,74]. Antimicrobial stewardship programs have a crucial role in limiting excess antibiotic use and providing expertise on extensively drug-resistant infections; however, the treatment of class B MBLs remains challenging.

In critically ill patients with sepsis, the pharmacokinetics (PK) is severely altered and leads to high inter- and intra-patient variability in dosing requirements. The PK of hydrophilic antibiotics such as β-lactams, aminoglycosides, or colistin is particularly impaired, as their volumes of distribution (Vd) are greatly increased in sepsis and septic shock [75]. Hypoalbuminemia is frequent and may increase the clearance of highly bounded antibiotics by increasing their unbound fraction. Consequently, antibiotic treatment underdosing is frequent, with up to 65% of critically ill patients receiving β-lactams not achieving maximal bacterial killing [76].

That is why the optimization of PK is instrumental, especially at the beginning of therapy. Some simple rules displayed in Table 2 favor an appropriate initial therapy. The glomerular hyperfiltration, common during the first phase of infection, should be taken into account. It should be kept in mind that traditional formulas for the estimation of creatinine clearance are not appropriate for critically ill patients, and thus the measurement of urine output and urine creatinine is required for an appropriate evaluation. The variability of volume of distribution and of clearance is important in the most severe patients, and therapeutic drug monitoring is probably preferable to optimize therapy [77].

In the fight against antimicrobial resistance, interventions to limit antibiotic exposure target the inappropriate use of antimicrobials, including excessive treatment duration. An unjustified prolonged antibiotic course also leads to higher health costs and a higher risk of antibiotic-related adverse events. On the other hand, inappropriate shortening of antibiotic therapy may be associated with a higher risk of treatment failure, especially if pharmacokinetic targets are difficult to reach, such as the treatment of challenging organisms. The appropriate treatment duration of MBL infections is not known [78]. Some simple rules may be helpful to individualize the duration of therapy in MBL infections. First of all, infection with a MBL-producing *Enterobacterales* is not a reason per se to prolong the duration of antibiotic therapy [79]. Second, although short therapy (5–7 days) is always preferable, it has been safely used only in the absence of underlying immune suppression, and requires an appropriate source control. Third, a short therapy should be safely used only if the clinical situation is stabilized with an improvement of signs of infections and the recovery of organ dysfunctions. Fourth, if bactericidal beta-lactam pivotal therapy is not an option, and therapy is based on colistin- or tigecycline-based regimens, available data may suggest that a short course is associated with more therapeutic failures [80].

Adapting antibiotic treatment duration based on the patient’s status could be a way to decrease overall antibiotic use, without compromising the safety of each individual treatment. Such algorithms for individualized treatment interruption have been evaluated, based on the evolution of clinical and biological variables. In a randomized trial on patients with GNB bloodstream infection, a C-reactive protein (CRP)-guided treatment was non-inferior to a 7-day or 14-day fixed treatment in terms of clinical failure rate [81]. The decrease in procalcitonin levels has also been successfully used to reduce antibiotic exposure in severe ICU patients [82].

## 9. Conclusions

MBL infections are increasingly common, even in non-endemic areas. The treatment should follow simple rules of antibiotic stewardship. Bactericidal therapy including new agents such as CZA/ATM or cefiderocol is effective. The treatment should be optimized through close collaboration with microbiologists and pharmacists, with determination of MICs to the available antimicrobials and a thorough therapeutic drug monitoring.

## Figures and Tables

**Table 1:** Cases series of severe NDM infections treated with CZA/ATM in ICU patients—experience of Bichat-Claude Bernard hospital.

Age, (Year), Gender	Medical History	SAPS II	SOFA Score (Treatment)	Invasive Ventilation	Shock	HD/CVVH	Source	Germ/MIC of CZA/ATM	Ttreatment Duration (Days)	Combo	Clinical Cure	Microbiological Cure	Survival (Hospital)	Cause of Death
76, Female	Obese; DiabetesARDS SARS-Cov2	42	2	Yes	No	No	VAP	Esherichia coli	1	Colistine	Yes	Yes	Alive	
42, Male	Obese, Diabetes, ARDS SARS-Cov2	46	10	Yes	Yes	Yes	VAP	Enterobacter cloacae; 0.064 mg/L	6		Yes	Yes	Death	Coma
58, Male	Endocarditis, mitral valve replacement	53	4	Yes	Yes	No	Septic shock in NDM colonized patient	Citrobacter freundii	2		Yes	Yes	Alive	
67, Female	renal transplant; hemorragic shock	47	10	No	No	No	BSI	Klebsiella pneumonia; 0.032 mg/L	15		No	Yes	Alive	
44, Female	lung transplant; acute respiratory failure	27	5	Yes	Yes	No	VAP	Klebsiella pneumoniae: 0.064 mg/L	52	Tigecycline	Yes	Yes	Alive	
53, Male	intraventricular communication/Endocarditis	40	9	Yes	Yes	Yes	Petitonitis; cellulitis	Echerichia coli; 0.094 mg/L (+ESBLE Klebsiella pneumoniae);	24	Colistine	Yes	Yes	Death	Shock
40, Female	Myocarditis, ECMO	34	8	Yes	Yes	Yes	SSI (ECMO cannulas)	Klebsiella pneumoniae; 0.38 mg/L	10		Yes	Yes	Death	Shock
36, Male	ARDS, SARS Cov2	23	3	Yes	No	No	VAP	Klebsiella pneumoniae; 0.064 mg/L	9		Yes	Yes	Alive	
70, Male	Chronic renal failure; Cardiac surgery (mitral valve replacement, tamponnade)	54	6	Yes	Yes	No	VAP	Enterobacter cloacae; 0.064 mg/L	9		No	Yes	Death	MOF

Abbreviations: VAP: ventilator-associated pneumonia; MOF: multiple organ failure; EBLSE: extended spectrum beta-lactamase *Enterobacterales*. ECMO: extra-corporeal membrane oxygenation; ARDS: acute respiratory distress syndrome; SSI: surgical site infection; BSI: bloodstream infection.

**Table 2:** Appropriate therapy of severe MBL infections: the 12 labors of physicians.

1. Do not treat simply colonized patients.
2. Use a pivotal beta-lactam antibiotic therapy with either the combination of aztreonam and ceftazidime-avibactam or cefiderocol.
3. A combination with another effective antimicrobial (Colistine, tigecycline, aminoglycoside, fosfomycin) is preferable before the knowledge of the susceptibility profile.
4. Ask the microbiological lab for MICs for the susceptible micro-organism.
5. A prolonged dual active antibiotic therapy is not recommended unless the use of a pivotal beta-lactam antibiotics is not possible. For colistin based antimicrobial regimen a combination therapy with another effective antibiotic is recommended.
6. The initial antibiotic dose should not be adapted to the renal clearance during the first 24 to 48 h of therapy.
7. For beta-lactam antibiotics, prolonged or continuous infusion should be used to improve the PK/PD.
8. In pneumonia, adjunctive nebulized antibiotic may be considered if not contra-indicated.
9. Monitor the creatinin clearance during therapy.
10. Therapeutic drug monitoring is important to optimize therapy and avoid over and under-dosage.
11. The duration of therapy should follow the guidelines for each infection. The individualization of the duration of therapy should depend on underlying illness, source control, the bactericidal nature of the pivotal antimicrobial and the improvement of clinical and biological parameters.
12. Protect the other patients. Antibiotic stewardship should be combined with strict infection control practices to avoid cross-transmissions of MBL *Enterobacterales*.
